# Association of lower liver function with cognitive impairment in the Shenzhen ageing-related disorder cohort in China

**DOI:** 10.3389/fnagi.2022.1012219

**Published:** 2022-10-13

**Authors:** Kaiyu Wu, Chunyan Xu, Guozhen Qiu, Qiwen Guo, Chunchun Chen, Wei Liu, Jianjun Liu, Kangding Liu, Feiqi Zhu

**Affiliations:** ^1^Department of Neurology, The First Hospital of Jilin University, Changchun, Jilin, China; ^2^Cognitive Impairment Ward of Neurology Department, The Third Affiliated Hospital of Shenzhen University Medical College, Shenzhen, Guangdong, China; ^3^Key Laboratory of Modern Toxicology of Shenzhen, Shenzhen Medical Key Subject of Health Toxicology (2020-2024), Shenzhen Center for Disease Control and Prevention, Shenzhen, Guangdong, China

**Keywords:** cognitive impairment, liver function, albumin, triglyceride, aspartate aminotransferase to alanine aminotransferase ratio

## Abstract

**Background:**

Accumulating evidence suggests that alterations in liver function may play an important role in the pathogenesis of Alzheimer’s disease (AD). However, it remains unclear whether there is any relationship between lower liver function and cognitive impairment among the elderly.

**Methods:**

From 2017 to 2018, we recruited 7,201 older people (over 60 years old) from 51 community health centers in the Luohu District of Shenzhen City. According to the Mini-Mental State Examination (MMSE) score and education level, participants were divided into a cognitive impairment group (*n* = 372) and a normal cognitive function group (*n* = 6,829). Nonparametric test, chi-square tests, and binary logistic regression were used to analyze the data.

**Results:**

Cognitive impairment group exhibits older age, more female sex, lower education level, and lower levels of albumin and triglyceride. Additionally, the aspartate aminotransferase (AST) to alanine aminotransferase (ALT) ratio was mainly distributed in the range of 1.17 to 1.3 in the cognitive impairment group, and 0.85 to 1.00 in the normal cognitive function group (*χ*2 = 10.02, *p* = 0.04). Binary logistic regression showed that cognitive impairment was significantly associated with age (*OR* = 0.934, 95%*CI*: 0.886–0.985, *p* = 0.017), female sex (*OR* = 2.255, 95%*CI*: 1.761–2.888, *p* < 0.001), lower education level (less than senior high school) (*OR* = 11.509, 95%*CI*: 9.064–14.613, *p* < 0.001), and lower albumin (*OR* = 1.023, 95%*CI*: 1.004–1.043, *p* = 0.011).

**Conclusion:**

Except for age, female sex, and lower education level, lower level of albumin and elevated AST to ALT ratio correlate with cognitive impairment. Whether lower liver function plays a role in AD needs to be further studied.

## Introduction

Senile dementia is a clinical syndrome characterized by substantial and progressive cognitive impairment and severe neurobehavioral symptoms that have an evident functional impact on daily life. Alzheimer’s disease (AD) accounts for 50–70% of all types of senile dementia (Jia et al., 2014; Wang et al., 2019). It is a chronic neurodegenerative disorder that affects millions of people worldwide and the number is expected to continue to grow with the aging of the population. The prevalence of AD in patients over 65 years of age is approximately 5%. It is estimated that nearly half of people aged over 85 will have AD (McDade and Bateman, 2017). Therapeutic developments are now focused on the early diagnosis of dementia at preclinical stages before the appearance of clinical symptoms (Lane et al., 2018; Zhou et al., 2019; Sayed et al., 2020).

In fact, a series of pathological changes started decades before the onset of dementia symptoms. Once symptoms of dementia became noticeable, the disease’s pathological alterations have been indicated to reach already an advanced stage (Reiman et al., 2011). It is known that the cardinal pathological features of AD are amyloid plaques and neurofibrillary tangles (Lane et al., 2018). Therefore, for decades, anti-amyloid and tau-aggregation inhibitors are the main therapeutic strategies. Nonetheless, the application of these methods in clinical practice has not been able to improve the management of this disease, as development strategies based on tau and Aβ have been declared as failures. Hence, the hypothesis of anti-amyloid and tau-aggregation inhibition has attracted criticism.

Interestingly, accumulating clinical and preclinical evidence indicates that AD is a metabolic disorder and that early metabolic alterations may contribute to a higher risk of dementia (Herholz et al., 2018). Several metabolic pathways have been found to be involved in AD pathogenesis, including inflammation, immune response, oxidative stress, and lipid metabolism (Mapstone et al., 2020). Although more attention has been paid to brain metabolism, these alterations might also get beyond the brain and affect metabolism in peripheral organs. Metabolic activities in the liver determine the state of the metabolic readout in the peripheral circulation (Zheng et al., 2019). Moreover, it has been suggested that AD is a systemic metabolic disorder, and it has been demonstrated that the liver is the earliest organ affected in APP/PS1 mice Transgenic (Tg) mice during amyloid pathology progression (Zheng et al., 2019). The liver is the main organ that metabolizes more than 60% of Aβ and decreased liver Aβ metabolism might result in the brain accumulation of Aβ (Maarouf et al., 2018). Therefore, hepatic metabolic abnormalities could reflect amyloid pathology progression. Previous research proposed that hepatic malfunction contributes to AD in a plethora of possible pathways, including the failure to maintain Aβ homeostasis at the periphery, thus providing a source of pro-inflammatory cytokines following chronic inflammation or injury, or metabolic impairment (Estrada et al., 2019). Accordingly, *Withania somnifera* has been shown to have a remarkable therapeutic effect and can reverse AD pathology by enhancing low-density lipoprotein receptor-related protein in the liver (Sehgal et al., 2012). A recent Framingham study revealed that middle-aged and older adults with a high risk of advanced liver fibrosis exhibit poorer cognitive function (Weinstein et al., 2019). Interestingly, Zhang et al. showed that HDL-C, triglyceride, and glucose levels measured in early to middle adulthood are significantly associated with incident AD several decades later, based on the results of the MMSE exam of Framingham Heart Study participants (Zhang et al., 2022). Similarly, a recent cohort study revealed that decreased liver functions, including lower levels of alanine aminotransferase (ALT) and aspartate aminotransferase (AST), were associated with AD diagnosis, impaired memory, and executive function, as well as cerebrospinal fluid biomarkers of AD, such as Aβ42 and p-Tau181 (Nho et al., 2019). In our previous study, we found that the liver weights of Tg mice decreased significantly and liver cell necrosis and lymphocyte infiltration increased significantly, however, the decreasing of liver weights and the increasing of liver cell necrosis and lymphocyte infiltration of Tg mice reversed significantly after accepting the parabiosis surgeries with wild type mice for 8 months (Kaiyu et al., 2022). In the present study, we aimed to investigate the association between liver function and cognitive impairment in the Shenzhen aging-related disorder cohort study in China.

## Materials and methods

### Study population

**Figure 1:**
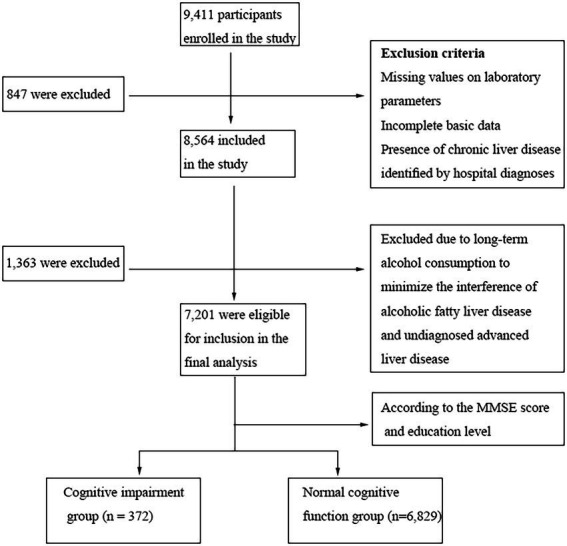
Flow diagram of this cross-sectional study.

In this study, we enrolled 9,411 participants aged 60 years and above from 51 community health centers in Luohu District, Shenzhen City, from 2017 to 2018. A total of 847 participants were excluded due to missing laboratory parameter values, incomplete basic data, and the presence of chronic liver disease identified by hospital diagnoses. At the same time, 1,363 participants with long-term alcohol consumption were excluded to minimize the interference of alcoholic fatty liver disease and undiagnosed advanced liver diseases. Finally, 7,201 participants were eligible for the final analysis. Written informed consent was obtained, which included permission for the analysis and data sharing. This study was approved by each participating site’s institutional review board. Trained physicians collected the clinical data *via* face-to-face interviews, which included demographic characteristics (age, sex, education level, weight, height, etc.), lifestyle characteristics (living alone, long-term alcohol intake, etc.), medical history (chronic hepatitis mainly), and mini-mental state examination (MMSE) assessment. According to the MMSE score and education level (education level was categorized as: (1) less than senior high school; (2) completed senior high school, vocational school, college, professional school, or graduate), subjects with MMSE score ≤ 21 (for subjects with an education level of primary school or below) or MMSE score ≤ 24 (for subjects with an education level of secondary school and above) were considered to have cognitive impairment. The 7,201 participants were then accordingly divided into a cognitive impairment group (*n* = 372) and a normal cognitive function group (*n* = 6,829; Figure 1).

### Clinical variables

Routine laboratory analyses were performed, and parameters including AST, ALT, albumin (ALB), total bilirubin (TB), blood lipid indices (total cholesterol [TC], triglyceride [TG], high-density lipoprotein cholesterol [HDL-C], and low-density lipoprotein cholesterol [LDL-C]) were evaluated. The TB-to-ALB ratio (TB/ALB) and AST-to-ALT ratio (AST/ALT) were calculated.

### Statistical evaluation

The Kolmogorov–Smirnov test was used to assess the normality of the continuous variances. Participant characteristics were summarized as means (standard deviation) or medians (first quartile, third quartile) for continuous data (depending on their distribution), and counts (proportions) for categorical variables. Continuous data were analyzed by an independent sample t-test when there was a normal distribution and by the Mann–Whitney test if there was a skewed distribution. Categorical data were analyzed using the chi-square test. The risk of cognitive impairment was assessed using binary logistic regression analysis. We modeled part of the continuous variables (e.g., AST, ALT, AST/ALT, etc.) in quintiles based on the data of participants enrolled to explore the correlation. The statistical significance level was set at *p* < 0.05 with a two-tailed distribution using SPSS statistical software (version 26.0; SPSS, Inc., Chicago, IL, United States).

## Results

Compared to the normal cognitive function group, the cognitive impairment group exhibited a higher age (68.00 [64.00, 75.00] vs. 67.00 years [63.00, 71.00], *p =* 0.001), fewer women (59.10% vs. 64.50%, *p* < 0.001), higher proportion of individuals living without a partner (15.9% vs. 14.2%, *p* < 0.001), and lower education level (57.8% vs. 43.4%, *p* < 0.001; Table 1). The levels of ALB and TG in the cognitive impairment group were significantly lower than those in the normal cognitive function group (44.10 [42.70, 45.68] vs. 44.60 [43.30, 45.90], *p* < 0.001) and (1.28 [0.90, 1.81] vs. 1.38 [0.98, 1.98], *p* = 0.003), respectively (Table 1). There were no significant differences between the two groups with respect to the body mass index (BMI), total cholesterol, LDL-C, HDL-C, TB, AST, ALT, AST to ALT ratio (AST/ALT), and TB-to-ALB ratio (TB/ALB). However, there was a significant difference in the distribution of the AST to ALT ratio by quintile grouping and chi-square test. In these conditions, the results showed that the distribution of the AST to ALT ratio in the cognitive impairment group ranged from 1.17 to 1.39, while in the cognitively normal group the it ranged from 0.85 to 1.00 (*c*^2^ = 10.02, *p* = 0.04) (Table 2). Binary logistic regression showed that older age (odds ratio (*OR*) 1.023, 95% confidence interval (*CI*): 1.004–1.043, *p* = 0.017), female sex (*OR* 2.255, 95% *CI*: 1.761–2.888, *p* < 0.001), educational level less than senior high school (*OR* 11.509, 95% *CI*: 9.064–14.613, *p* < 0.001), and lower albumin level (*OR* 1.066, 95% *CI*: 1.013–1.122, *p* = 0.014) were independently associated with cognitive impairment (Table 3).

**Table 1:** Demographics and laboratory parameters of the study population.

Parameters	Cognitive impairment subjects	Normal cognitive function subjects	*p*-value
	(*n* = 372)	(*n* = 6,829)	
Age (years)	68.00 (64.00,75.00)	67.00 (63.00,71.00)	0.001^*^
Sex (female)	59.10%	64.50%	<0.001^*^
Education (less than senior high school)	57.80%	43.40%	<0.001^*^
Living alone	15.90%	14.20%	<0.001^*^
BMI (kg/m^2^)	24.08 (22.30,26.00)	24.24 (22.28,26.32)	0.260
AST(U/L)	20.00 (17.00,24.00)	20.00 (17.00,24.00)	0.430
ALT(U/L)	17.00 (14.00,24)	18.00 (14.00,25.00)	0.130
AST/ALT	1.12 (0.89,1.36)	1.08 (0.88,1.31)	0.180
TC (mmol/L)	5.45 (4.65,6.22)	5.50 (4.80,6.22)	0.180
TG (mmol/L)	1.28 (0.90,1.81)	1.38 (0.98,1.98)	0.003^*^
LDL-C (mmol/L)	3.12 (2.50,3.73)	3.13 (2.56,3.72)	0.470
HDL-C (mmol/L)	1.51 (1.30,1.76)	1.51 (1.29,1.77)	0.970
ALB (g/L)	44.10 (42.70,45.68)	44.60 (43.30,45.90)	0.001^*^
TB (μmol/L)	14.70 (12.00,17.80)	14.80 (12.00,18.20)	0.270
TB/ALB (μmol/g)	0.32 (0.26,0.39)	0.33 (0.27,0.41)	0.480

BMI, body mass index (calculated as kg/m^2^); AST, aspartate aminotransferase; ALT, alanine aminotransferase; AST: ALT, AST to ALT ratio; TC, total cholesterol; TG, triglyceride; LDL-C, low-density lipoprotein cholesterol; HDL, high-density lipoprotein cholesterol; ALB, albumin; TB, total bilirubin; TB/ALB, TB to ALB ratio.

* Statistically significant differences at *p* < 0.05 (2-tailed).

**Table 2:** Chi square test analysis results of the different range of AST/ALT.

	AST/ALT range^*^
Group	≤0.844	0.845–1.000	1.001–1.167	1.168–1.385	≥1.386
Cognitive impairment (%)	19.10	21.20	13.20	23.40	23.10
Normal cognition (%)	20.10	22.70	18.00	19.40	19.80

AST, aspartate aminotransferase; ALT, alanine aminotransferase.

* Statistically significant differences at *p* < 0.05 (2-tailed).

**Table 3:** Multivariate logistic regression analysis for demographics and laboratory parameters of the study population.

Variables	Univariate		Multivariate	
	*OR* (95%*CI*)	*p-*value	*OR* (95%*CI*)	*p*-value
Age	1.058 (1.040–1.077)	<0.001^*^	1.023 (1.004–1.043)	0.017^*^
Sex (female vs. male)	1.229 (0.994–1.521)	0.050^*^	2.255 (1.761–2.888)	<0.001^*^
Education level (less than senior high school)	9.349 (7.519–11.626)	<0.001^*^	11.509 (9.064–14.613)	<0.001^*^
BMI	0.984 (0.952–1.017)	0.340		
TG	0.910 (0.814–1.019)	0.102		
ALB	0.913 (0.870–0.959)	<0.001^*^	0.934 (0.886–0.985)	0.011^*^
AST/ALT	1.155 (0.871–1.532)	0.318		

BMI, body mass index (calculated as kg/m^2^); TG, triglyceride; ALB, albumin; AST, aspartate aminotransferase; ALT, alanine aminotransferase; AST/ALT, AST to ALT ratio.

* Statistically significant differences at *p* < 0.05 (2-tailed).

## Discussion

In the present study, we examined the association between liver function and cognitive impairment among 7,201 elderly individuals without any medical history of chronic liver disease or long-term alcohol consumption in the Shenzhen aging-related disorder cohort in China. The results of our indicate that advanced age, living alone, lower education level, lower levels of ALB and TG, and elevated AST to ALT ratio were associated with cognitive impairment among the elderly.

The AST to ALT ratio is an indicator of liver function and our results revealed that the AST to ALT ratio was higher in the cognitive impairment group than in the normal cognitive function group (Table 2). This is similar to the findings from a previous study, where AST to ALT ratio was found to be positively associated with AD diagnosis and poor cognitive performance among the subjects (Han et al., 2022). However, in contrast to the outcome of a previous study that reported an increased AST to ALT ratio and a lower ALT level in individuals with poor cognition (Nho et al., 2019), our study revealed no statistically significant differences in the levels of ALT between the two groups. A recent prospective study demonstrated that low levels of the plasma aminotransferases, AST and ALT, in mid-life, particularly ALT, were associated with an elevated long-term risk of dementia (Lu et al., 2021). Hepatocytes can act directly on circulating Aβ, promoting its clearance by degradation or through the bile excretion (Kanekiyo and Bu, 2014). Altered levels of aminotransferases are sensitive indicators of hepatocyte injury. Specifically, the concentration of ALT is the highest in hepatocytes but lower in other tissues, whereas AST is expressed in tissues of many other organs including muscle, kidney, and brain. Liver metabolic function could be reflected by the levels of aminotransferases, and altered liver enzymes lead to disturbances in liver-associated metabolites. Clinical researchers studying ALT and AST mostly focused on increases in their levels. However, decreased liver enzyme synthesis and metabolism (reflected by reduced plasma aminotransferase levels) may be associated with cerebral hypometabolism, which has been reported to occur before the onset of dementia (Clarke et al., 2018). A previous study claimed that disrupted brain energy metabolism could be one of the earliest hallmarks of AD (Zheng et al., 2018). It has also been verified that lower ALT levels and increased AST to ALT ratios are consistent with reduced brain glucose metabolism in humans (Nho et al., 2019). In addition, plasma AST and ALT have been shown to have a significantly positive correlation with plasma glutamate levels in healthy adult subjects (Kamada et al., 2016). Furthermore, both ALT and AST promote glutamate production, and glutamate acts as a neurotransmitter in neuronal cells, and this is related to the memory development (Cassano et al., 2016; Ribeiro et al., 2017). Glutamate is the major fast excitatory neurotransmitter and is involved in almost all CNS functions, especially in cortical and hippocampal regions related to learning and memory, and about 70% of all excitatory synapses in the CNS utilize glutamate as a neurotransmitter (Parsons et al., 1998).

Interestingly, our finding revealed a lower ALB level in the cognitive impairment group compared to the normal cognition group (44.1 [42.7, 45.68] vs. 44.6 [43.4, 45.9] g/L, *p* = 0.001; Table 1). This is concordant with results from the Longitudinal Aging Study Amsterdam, in which an association between low serum ALB and cognitive function decline was observed in 1,284 older individuals (Serre-Miranda et al., 2020). Moreover, a recent study showed that intravenous supplementation with human ALB improved daily function and reduced dementia burden in patients with AD (Zhong et al., 2020). Considering that AD is a multifactorial disease, there are two mechanisms that may explain the lower ALB level in the cognitive impairment group. First, ALB is considered to be the gold-standard marker of the protein synthesis function of the liver (Woreta and Alqahtani, 2014). It is a critical plasma protein and is regarded as one of the most potent Aβ sequestering systems in that it maintains the dynamic equilibrium of Aβ between the brain and blood plasma (Xie and Guo, 2020). A reduction in the serum ALB in the blood may lead to a decrease in the capacity of Aβ to shift from the brain to the blood, resulting in Aβ deposition in the brain (Stanyon and Viles, 2012). Second, findings from a study showed that ALB has a defensive role in the process of inflammation and infection (Ng et al., 2009), and substantial evidence suggests that inflammatory mechanisms are critical players in the pathogenesis of dementia, including AD (Holmes, 2013). Taken together, the lower levels of ALB reported in this study may indicate an increased risk of cognitive impairment.

Unexpectedly, as shown in Table 1, we found that the serum triglyceride levels in the cognitive impairment group were lower than those in the normal cognitive function group (1.28 [0.90, 1.81] vs. 1.38 [0.98, 1.98] mmol/L, *p* = 0.003). In agreement with our findings, some studies researchers reported that subjects with probable AD exhibit significantly lower serum triglyceride levels compared to the controls (Dimopoulos et al., 2007; Lepara et al., 2009; Lim et al., 2020). However, several other studies reported no correlation between blood triglyceride levels and memory and cognitive abilities (van Exel et al., 2002; Henderson et al., 2003; Dimache et al., 2021). The liver is recognized as an important site of lipid metabolism, including lipid digestion, absorption, synthesis, decomposition, and transport. In the present cross-sectional study, among the lipid profile, only serum triglyceride levels were found to be lower in the cognitive impairment group compared to the normal cognitive function group. Moreover, BMI was not significantly different between the two groups, which suggests that the decrease in the serum triglyceride levels in the cognitive impairment group might not be due to poor nutritional status. As epidemiological studies exploring the association between serum lipids and dementia have reported conflicting results, it is difficult to draw a firm conclusion on the relationship between triglyceride levels and the risk of AD development based on the current literature (Agarwal and Khan, 2020). So far, there is no clear explanation for the decreased triglyceride levels in the elderly with cognitive impairment.

Our study has some limitations that should be acknowledged. The major limitation of our study is its cross-sectional design; therefore, we could not establish any cause-effect relationship. Also, although the MMSE is a standard measure of global cognitive function, participants with MMSE scores of 24 or above may include some cases with subclinical or mild cognitive impairment and early dementia. Additionally, the time of evaluation of cognitive impairment may not exactly reflect the disease onset. Finally, we analyzed a large representative sample of the Chinese elderly population, allowing for the complex sampling design, and thus our results may not be generalizable to more ethnically diverse populations. While we controlled for some potential confounders, the possibility of residual confounding factors still exists.

In conclusion, our findings suggest that elevated AST to ALT ratio and decreased levels of albumin and plasma triglyceride are associated with cognitive impairment, supporting the hypothesis that liver function alterations are involved in AD pathogenesis. Additional prospective, long-term, and follow-up cohort clinical studies may be needed to address this critical issue and confirm the findings.

## Data availability statement

The raw data supporting the conclusions of this article will be made available by the authors, without undue reservation.

## Ethics statement

The studies involving human participants were reviewed and approved by the Independent Ethics Committee of the Third Affiliated Hospital of Shenzhen University Medical College. The patients/participants provided their written informed consent to participate in this study.

## Author contributions

FZ designed the study, analyzed the data, interpreted the data, and took the lead in writing the manuscript. KW analyzed the data, interpreted the data, and wrote the manuscript. CX, GQ, QG, and CC collected the data and accomplished MMSE assessments. WL and JL designed the study and collected the data. KL analyzed and interpreted the data. All authors contributed to the article and approved the submitted version.

## Funding

This work was supported by Sanming Project of Medicine in Shenzhen (SZSM201801014) and Key project of Shenzhen Science and Technology Commission (JCYJ20200109143431341).

## Conflict of interest

The authors declare that the research was conducted in the absence of any commercial or financial relationships that could be construed as a potential conflict of interest.

## Publisher’s note

All claims expressed in this article are solely those of the authors and do not necessarily represent those of their affiliated organizations, or those of the publisher, the editors and the reviewers. Any product that may be evaluated in this article, or claim that may be made by its manufacturer, is not guaranteed or endorsed by the publisher.
